# Studies on Chromatographic Fingerprint and Fingerprinting Profile-Efficacy Relationship of *Saxifraga stolonifera* Meerb.

**DOI:** 10.3390/molecules201219882

**Published:** 2015-12-19

**Authors:** Xing-Dong Wu, Hua-Guo Chen, Xin Zhou, Ya Huang, En-Ming Hu, Zheng-Meng Jiang, Chao Zhao, Xiao-Jian Gong, Qing-Fang Deng

**Affiliations:** 1 Guizhou Engineering Laboratory for Quality Control & Evaluation Technology of Medicine, Guizhou Normal University, 116 Baoshan North Rd., Guiyang 550001, Guizhou, China; wuxingdong2013@sina.com (X.-D.W.); huaguochen81@gmail.com (H.-G.C.); huangya2014056@sina.com (Y.H.); 47780hu@sina.com (E.-M.H.); 13555711040@163.com (Z.-M.J.); chaozhao@126.com (C.Z.); gongxiaojian1@163.com (X.-J.G.); dqf_2012@sina.com (Q.-F.D.); 2 The Research Center for Quality Control of Natural Medicine, Guizhou Normal University, 116 Baoshan North Rd., Guiyang 550001, Guizhou, China; 3 Guiyang College of Traditional Chinese Medicine, 50 Shidong Rd., Guiyang 550002, Guizhou, China

**Keywords:** *Saxifraga stolonifera*, HPLC fingerprints, anti-benign prostatic hyperplasia activities, spectrum–effect relationships, chemometrics

## Abstract

This work investigated the spectrum-effect relationships between high performance liquid chromatography (HPLC) fingerprints and the anti-benign prostatic hyperplasia activities of aqueous extracts from *Saxifraga stolonifera*. The fingerprints of *S. stolonifera* from various sources were established by HPLC and evaluated by similarity analysis (SA), hierarchical clustering analysis (HCA) and principal component analysis (PCA). Nine samples were obtained from these 24 batches of different origins, according to the results of SA, HCA and the common chromatographic peaks area. A testosterone-induced mouse model of benign prostatic hyperplasia (BPH) was used to establish the anti-benign prostatic hyperplasia activities of these nine *S. stolonifera* samples. The model was evaluated by analyzing prostatic index (PI), serum acid phosphatase (ACP) activity, concentrations of serum dihydrotestosterone (DHT), prostatic acid phosphatase (PACP) and type II 5α-reductase (SRD5A2). The spectrum-effect relationships between HPLC fingerprints and anti-benign prostatic hyperplasia activities were investigated using Grey Correlation Analysis (GRA) and partial least squares regression (PLSR). The results showed that a close correlation existed between the fingerprints and anti-benign prostatic hyperplasia activities, and peak 14 (chlorogenic acid), peak 17 (quercetin 5-*O*-β-d-glucopyranoside) and peak 18 (quercetin 3-*O*-β-l-rhamno-pyranoside) in the HPLC fingerprints might be the main active components against anti-benign prostatic hyperplasia. This work provides a general model for the study of spectrum-effect relationships of *S. stolonifera* by combing HPLC fingerprints with a testosterone-induced mouse model of BPH, which can be employed to discover the principle components of anti-benign prostatic hyperplasia bioactivity.

## 1. Introduction

Traditional Chinese medicines (TCMs) have attracted more and more attention in recent years since they exhibit weak toxicity, affordability and complementary therapeutic effects against many diseases, and many of them were reported to have the anti-benign prostatic hyperplasia biological activities [1]. Recent studies have shown that the extract of *Saxifraga stolonifera* Meerb. has a strong effect on the treatment of benign prostatic hyperplasia (BPH) [2]. The main phytochemical constituents of *S. stolonifera* are flavonoids, organic acids and phytosterols such as bergenin, gallic acid and β-sitosterol [3]. Pharmacological experiments have indicated that extracts of *S. stolonifera* have a wide range of biological activities, including bacteriostatic [4], antioxidant [4,5] and antitumor activities, *etc.* [2].

As is known to us, BPH is one of the most common urinary disorders in the aging male. The prevalence of histologically identifiable BPH was characterized by hyperplasia of the prostate stromal as well as epithelial cells, which results in prostate gland enlargement [6]. Numerous studies have reported that the prevalence of BPH was greater than 50% in men of 60 years and reaches approximately 90% by the age 85 [7,8]. The symptoms of BPH include urinary frequency, weak urine stream, nocturia and lower urinary tract symptoms (LUTS) [9], which can cause significant impact on the quality of life. Many kinds of medicines are prevalent in treating BPH, such as hormonal therapy, alpha 1-adrenoreceptor blockers and 5 alpha-reductase inhibitors [10]. The finasteride has been used for many years in the treatment of BPH and it reduced local production of the growth promoted by androgen dihydrotestosterone (DHT) [11,12]. However, the onset of finasteride-related adverse effects was often associated with these options such as erectile dysfunction, fatigue, dizziness, ejaculatory dysfunction and upper respiratory tract infection [9,13]. Therefore, searching for more natural plant extracts for the management of these conditions has attracted much attention [14,15,16].

Chromatographic fingerprint was accepted as a useful method for the identification and quality control of traditional Chinese medicine in recent years [17,18], but it cannot be employed to identify the components which play the leading role in treatment. So, it is imperative and urgent to investigate the spectrum-effect relationship between HPLC fingerprints and efficacy, establish integrated evaluation system and finally find the principal bioactive components in the fingerprint representing the curative effect [19].

Water decoction is a classical extract method that has been widely accepted in Chinese folk since his convenience, environmental protection [20,21]. Then, water decoction was chosen to extract *S. stolonifera* samples. In the present study, HPLC was applied to establish the fingerprints of *S. stolonifera* aqueous extract from different regions, and investigate the curative effectiveness of the aqueous extract of *S. stolonifera* (*AESS*) in a testosterone-induced BPH model in castrated mice. According to the results of *S. stolonifera* fingerprints and pharmacodynamics activities, partial least squares regression (PLSR) and Gray Correlation Analysis (GRA) were used to learn the primary active components against benign prostatic hyperplasia.

## 2. Results and Discussion

### 2.1. HPLC Fingerprints

#### 2.1.1. Analysis of HPLC Fingerprints and Similarities 

**Figure 1:**
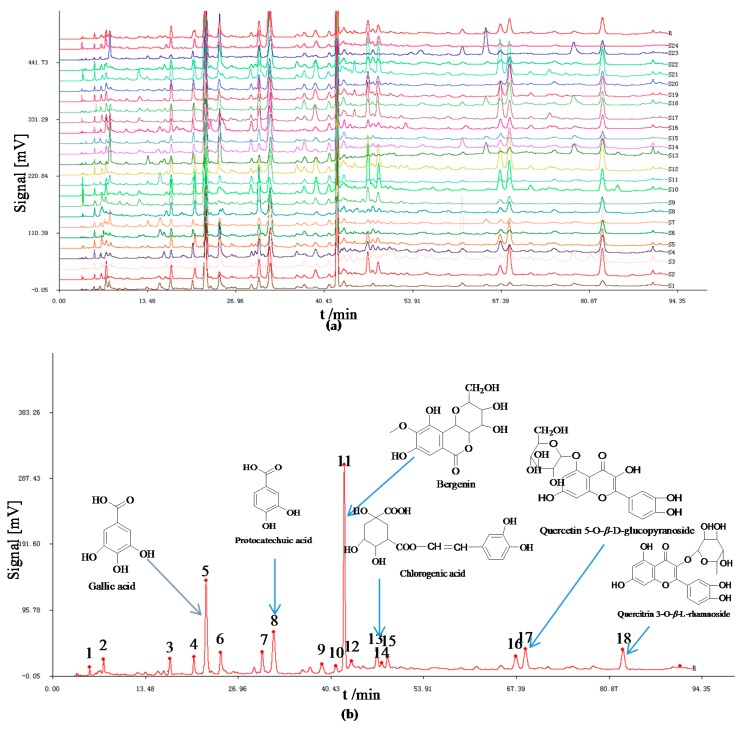
(**a**) The high performance liquid chromatography (HPLC) fingerprints of aqueous extracts of *S. stolonifera* from various sources; (**b**) The reference chromatogram was generated from the fingerprints of the 24 batches of *S. stolonifera* samples by using the Similarity Evaluation System for Chromatographic Fingerprint of traditional Chinese medicines (TCM) using the median method.

Our previous studies of methodology validation showed that the method of HPLC for the fingerprint analysis had good segregation from consecutive peaks and with large areas. As the Figure 1a showed the typical HPLC fingerprints of *AESS* from 24 batches and reference standard fingerprint was generated at the same time Figure 1b. Eighteen common peaks were found in the reference chromatogram of *S. stolonifera* by comparison of their HPLC retention time, most the chromatogram shapes of congeneric sample from different sources were quite similar. However, there were still some fingerprint differences among these samples of different sources, such as the values of peak area and peak number (which is equal to the injection volume) shown in Figure 1a. The state food and drug administration (SFDA) of China advocated that all herbal chromatograms should be evaluated in terms of similarity by calculation of the correlative coefficient and/or angle cosine values of original data [22,23]. The similarities between the entire chromatographic profiles of 24 batches of *S. stolonifera* and the reference chromatogram were evaluated by SA, and correlation coefficients of their chemical fingerprints were shown in Table 1. The results showed that correlation coefficients of those samples were from 0.027 (Qingchang town, Guizhou, China) to 0.990 (Qitan town, Guizhou, China), and the correlation coefficients between the chromatograms of samples from the same source there were very different.

#### 2.1.2. Results of HCA

**Figure 2:**
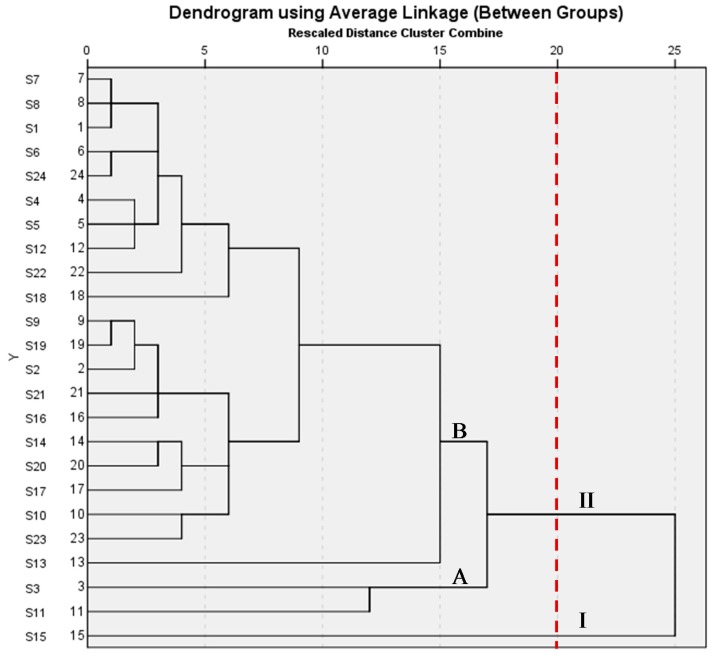
Hierarchical clustering analysis of *S. stolonifera* samples. The hierarchical clustering analysis (HCA) of samples 1–24 was performed using SPSS statistics software (SPSS for Windows 20.0, SPSS Inc., New York, NY, USA). A method called Between-Groups Linkage was applied. І and II represent cluster I and cluster II, respectively. A and B represent subgroup A and subgroup B, respectively.

In order to assess this tendency, a hierarchical agglomerative cluster analysis of samples was performed. HCA carried out were generating different clusters according to similarity of fingerprints. Between group methods, one of the most efficient methods for the analysis of variance between clusters was applied, and Square Euclidean distance was selected as a measurement. As the results in Figure 2 show, it was clear that 24 tested samples of *S. stolonifera* were divided into two main clusters. Sample no. **15** was in cluster I and the other samples were in cluster II which was divided into two subgroups again. Sample nos. **3** and **11** were in subgroup A and the others were in subgroup B. The result suggested the contents and distribution of the main components were different in different *S.* *stolonifera* samples, which would result in their different efficacies.

**Table 1:** Collected information and similarity of the samples.

Sample No.	Sources	Acquisition Time	Similarity	Sample No.	Sources	Acquisition Time	Similarity
**S1**	Haozhou city, Medicine Market, Anhui	May, 2014	0.984	**S13**	Kaiyang county, Medicine Market, Guizhou	May, 2014	0.949
**S2**	Baiyun town, Guizhou	April, 2014	0.978	**S14**	Censong town, Guizhou	April, 2014	0.979
**S3**	Qingchang town, Guizhou	June, 2014	0.027	**S15**	Majiangxiasi town, Guizhou	October, 2012	0.981
**S4**	Yanxia town, Guizhou	July, 2014	0.972	**S16**	Dujiangyan city Medicine Market, Sichuan	April, 2014	0.758
**S5**	Guilin city, Medicine Market, Guangxi	May, 2014	0.983	**S17**	Benzhuang town, Guizhou	March, 2014	0.956
**S6**	Qingping, Medicine Market, Guangdong	March, 2014	0.972	**S18**	Qitan town, Guizhou	April, 2014	0.990
**S7**	Huaguoyuan, Medicine Market, Guizhou	May, 2014	0.979	**S19**	Shidong town, Guizhou	March, 2014	0.983
**S8**	Dongfeng town, Guizhou	July, 2014	0.599	**S20**	Zhenfeng county, Medicine Market, Guizhou	April, 2014	0.986
**S9**	Shuitian town, Guizhou	March, 2014	0.935	**S21**	Liutong town, Guizhou	March, 2014	0.942
**S10**	Guizhou, Botanical Garden, Guizhou	April, 2014	0.973	**S22**	Liutun town, Guizhou	July, 2014	0.981
**S11**	Baoding city, Medicine Market, Hebei	May, 2014	0.981	**S23**	Zhazuo town, Guizhou	July, 2014	0.941
**S12**	Gaopo town, Guizhou	March, 2014	0.970	**S24**	Banqiao town, Guizhou	June, 2014	0.967

#### 2.1.3. Results of PCA

Large data sets are becoming more common in our scientific research, and PCA has turned out to be an extremely useful tool to reduce the computation burden [24]. In this study, we considered 18 common peaks areas of 24 samples as research objects. Therefore, the 18 common peak areas of different resolved components were analyzed in different samples and a new data matrix with dimensions 24 samples × 18 variables (components) was developed. The peak areas of 18 components in 24 samples are the elements of this new data matrix. For multivariate classification of chromatographic fingerprints, this new data matrix was calculated by PCA. Auto-scaling was chosen as a preprocessing step before PCA analysis [25]. In order to obtain more accurate and detailed information about the fingerprints, SPSS software was used for PCA. The result of the PCA showed that first six principal components (Z_1_ to Z_6_) contained 82.49% of the information of the original 18 indexes. The total variance explained in Table 2 shows the distribution of these 18 parameters. The 18 relations of first six components referencing to the Eigenvectors is as follows:

Z_1_ = 0.01x_1_ − 0.12x_2_ + 0.32x_3_ + 0.50x_4_ + 0.27x_5_ + 0.29x_6_ + 0.02x_7_ + 0.04x_8_ + 0.36x_9_ + 0.65x_10_ + 0.60x_11_ + 0.48x_12_ + 0.45x_13_ + 0.59x_14_ + 0.47x_15_ + 0.71x_16_ + 0.83x_17_ + 0.85x_18_ (1)

Z_2_ = −0.40x_1_ + 0.09x_2_ − 0.80x_3_ − 0.75x_4_ + 0.40x_5_ + 0.64x_6_ − 0.17x_7_ + 0.64x_8_ + 0.05x_9_ + 0.21x_10_ + 0.55x_11_ + 0.34x_12_ + 0.09x_13_ + 0.08x_14_ + 0.01x_15_ − 0.41x_16_ − 0.21x_17_ + 0.71x_18_ (2)

Z_3_ = −0.15x_1_ − 0.09x_2_ − 0.58x_3_ + 0.20x_4_ + 0.08x_5_ + 0.64x_6_ + 0.68x_7_ − 0.41x_8_ − 0.47x_9_ + 0.33x_10_ − 0.13x_11_ + 0.63x_12_ − 0.43x_13_ − 0.08x_14_ − 0.61x_15_ − 0.07x_16_ + 0.15x_17_ − 0.09x_18_ (3)

Z_4_ = −0.73x_1_ − 0.06x_2_ − 0.22x_3_ − 0.01x_4_ − 0.21x_5_ + 0.18x_6_ + 0.34x_7_ + 0.28x_8_ − 0.43x_9_ − 0.52x_10_ − 0.11x_11_ − 0.09x_12_ + 0.56x_13_ + 0.51x_14_ − 0.31x_15_ − 0.11x_16_ − 0.07x_17_ + 0.42x_18_ (4)

Z_5_ = −0.14x_1_ − 0.30x_2_ − 0.17x_3_ − 0.11x_4_ + 0.25x_5_ + 0.22x_6_ + 0.71x_7_ − 0.05x_8_ + 0.60x_9_ − 0.05x_10_ − 0.45x_11_ − 0.07x_12_ − 0.12x_13_ + 0.07x_14_ + 0.46x_15_ − 0.03x_16_ − 0.11x_17_ − 0.06x_18_ (5)

Z_6_ = −0.28x_1_ + 0.49x_2_ − 0.16x_3_ − 0.25x_4_ + 0.06x_5_ − 17x_6_ + 0.11x_7_ + 0.12x_8_ + 0.05x_9_ − 0.24x_10_ + 0.08x_11_ + 0.04x_12_ − 0.39x_13_ − 0.21x_14_ + 0.04x_15_ + 0.30x_16_ + 0.33x_17_ + 0.15x_18_ (6)

**Table 2:** Total Variance Explained.

Component	Initial Eigenvalues			Extraction Sums of Squared Loadings	
	Total	% of Variance	Cumulative %	Total	% of Variance
1	4.324	24.022	24.022	4.324	24.022
2	3.075	17.085	41.107	3.075	17.085
3	2.741	15.228	56.335	2.741	15.228
4	2.192	12.179	68.513	2.192	12.179
5	1.569	8.716	77.230	1.569	8.716
6	0.947	5.261	82.490	0.947	5.261
7	0.816	4.533	87.023	0.816	4.533
8	0.699	3.882	90.906	0.699	3.882
9	0.407	2.264	93.169	0.407	2.264
10	0.346	1.922	95.092	0.346	1.922
11	0.267	1.485	96.577	0.267	1.485
12	0.221	1.231	97.807	0.221	1.231
13	0.159	0.882	98.689	0.159	0.882
14	0.099	0.552	99.241	0.099	0.552
15	0.081	0.451	99.692	0.081	0.451
16	0.040	0.220	99.913	0.040	0.220
17	0.013	0.071	99.984	0.013	0.071
18	0.003	0.016	100.000	0.003	0.016

The absolute value of the coefficient before x_1_, x_2_, x_3_, …, x_17_ and x_18_ was the coefficient between the principal component and the 18th parameter. The bigger the coefficient of the parameter, the better the correlation the principal component had with the parameter. The Equations of (1)–(6) showed the values of Z_1_–Z_6_ (the first six principal components), which were mainly decided by x_1_, x_2_, x_3_, x_7_ and x_18_, showing that A_1_, A_2_, A_3_, A_17_ and A_8_ might the main influence in the Z_1_–Z_6_.

### 2.2. Results of Screening Differences Samples

On the basis of chromatographic fingerprints of the 24 batches of *S. stolonifera* and the chemometrics including similarity evaluation, PCA, and HCA, nine batches of *S. stolonifera* with different chemical profiles were selected for researches on their activities and for profile-efficiency study. The specific methods by which we chose these nine batches of *S. stolonifega* are as follows: the peaks of S2 (from Baiyun town, Douyun, China) have good resolution and therefore we selected it as the reference chromatogram when the HPLC fingerprint of other *S. stolonifera* extracts was established. According to the results of HCA, sample **S15** was classified in cluster I. Besides, on the basis of the cluster II, PCA and common peaks area, we selected the other seven samples. Therefore, we choose the nine samples of different origins as follows: **A** (**S2**) from Baiyun town, Anshun city, China; **B** (**S3**) from Qingchang town, Bijie city, China; **C** (**S4**) from Yanxia town, Duyun city, China; **D** (**S9**) from Shuitian town, Guiyang city, China; **E** (**S13**) from Kaiyang Medicine Market, Guiyang city, China; **F** (**14**) from Censong town, Kaili city, China; **G** (**S14**) from Majiangxiasi town, Kaili city, China; **H** (**S21**) from Liutong town, Guiyang city, China; **I** (**23**) from Zhazuo town, Guiyang city, China were using pharmacodynamics analysis.

### 2.3. Results of Anti-Benign Prostatic Hyperplasia Activities

#### 2.3.1. Effect of *AESS* on Prostate Index

The prostate index is an important indicator in BPH. The results were shown in Table 3. The PI showed a significant increase in the BHP model control group (51.52 ± 5.56 mg/100 g body weight, *p* < 0.01) compared with the *AESS* treated groups (**A**–**E**) (*p* < 0.01) and sample **G** (*p* < 0.05). The finasteride control group and QLKT control group showed a significantly lower PI (*p* < 0.01) compared to the BHP model control group. The results showed that the animal model used in this study was suitable for evaluating the effect of *AESS* on the growth of the prostate. Administration of the tested sample significantly reduced the PI of BPH in our mouse, and the effects were similar to the currently-used drugs finasteride and QLKT.

#### 2.3.2. Effect of Aqueous Extracts of *S. stolonifera* on Serum DHT Concentration

DHT is the product of the 5α-reduction of testosterone (T). The particular androgens were shown to be two or three times more potent than testosterone in target tissues. Because DHT could cause pathologic prostate growth, it can not only be detrimental in the adult prostate but also plays a beneficial role in the developing prostate [26]. Then, the level of DHT in serum was used to evaluate the effect of *AESS* in anti-benign prostatic hyperplasia. According to the result, the BHP model control group had significantly increased DHT levels (187.54 ± 29.75 nmol/L) compared with the control group (130.50 ± 15.41 nmol/L, *p* < 0.01). The Finasteride control group results were 126.47 ± 15.42 nmol/L, *p* < 0.01 and the QLKT control group results were 128.43 ± 18.00 nmol/L, *p* < 0.01. Administration of *AESS* from different habitats is shown in Table 3. Compared with the BHP model control group, all of the *AESS* from nine batches had a significantly (*p* < 0.01) lower serum DHT levels except both sample **H** and **I** (*p* < 0.05). This study confirmed that serum DHT concentration was significantly elevated in the mouse model of BPH and administration of the *AESS* all shows significantly reduces serum DHT concentration.

#### 2.3.3. Effect of *AESS* on Serum ACP Activity

ACP is produced in the liver, spleen and prostate gland and it has long been used as a clinical serum biomarker of BPH and prostate cancer [27]. Therefore, the activity of ACP on the serum was chosen as the index of BPH. From the data in Table 3, the serum ACP activity of the BPH model control group (69.79 ± 8.45 IU/L) was significantly higher than that of the control group (47.89 ± 5.90 IU/L, *p* < 0.01). The finasteride control group, at a dose of 1 mg/kg, significantly decreased the serum activity of ACP (46.63 ± 8.49 IU/L, *p* < 0.01) in castrated mice treated with testosterone, compared to the BPH model control group. Mice which received QKPT administered orally (750 mg/kg body weight), had significantly decreased ACP serum activity (50.60 ± 7.84 IU/L, *p* < 0.01). The serum activity of ACP in castrated mice treated with testosterone and administered *AESS* was obviously lower than the BPH model control group (all *p* < 0.01). All of the test samples from different habitats displayed significantly decreasing serum ACP activity and similar effects as in the finasteride control group.

**Table 3:** Effect of aqueous extracts of *S. stolonifera* on the serum acid phosphatase (ACP) activity, prostate index (PI), and the concentration of serum dihydrotestosterone (DHT), prostatic acid phosphatase (PACP) and SRD5A2 in a castration and testosterone-induced mice model of benign prostatic hyperplasia (BPH).

Groups	Prostatic Index (mg/100 g Body Weight)	Serum DHT Concentration (nmol/L)	Serum ACP Activity (IU/L)	Serum PACP Concentration (ng/L)	Serum SRD5A2 Concentration (pg/L)
control group	36.42 ± 2.36 ^##^	130.50 ± 15.41 ^##^	47.89 ± 5.90 ^##^	647.85 ± 54.63 ^##^	78.23 ± 9.26 ^##^
BPH model control group	51.52 ± 5.56	187.54 ± 29.75	69.79 ± 8.45	816.66 ± 60.85	125.23 ± 9.69
Finasteride control group	35.15 ± 5.33 ^##^	126.47 ± 15.42 ^##^	46.63 ± 8.49 ^##^	698.06 ± 32.38 ^##^	90.72 ± 9.54 ^##^
QKPT control group	36.11 ± 3.46 ^##^	128.43 ± 18.00 ^##^	50.60 ± 7.84 ^##^	675.98 ± 55.81 ^##^	89.86 ± 12.60 ^##^
group A	37.47 ± 4.67 ^##^	127.02 ± 14.80 ^##^	50.23 ± 4.89 ^##^	708.86 ± 40.00 ^##^	101.04 ± 13.99 ^##^
group B	37.22 ± 7.34 ^##^	133.97 ± 7.79 ^##^	54.23 ± 5.91 ^##^	750.58 ± 47.51 ^#^	105.10 ± 6.61 ^#^
group C	38.57 ± 5.84 ^##^	153.06 ± 28.35 ^##^	55.18 ± 5.39 ^##^	878.04 ± 50.05 ^#^	106.87 ± 19.10 ^#^
group D	38.09 ± 6.07 ^##^	125.02 ± 13.57 ^##^	48.76 ± 5.46 ^##^	755.04 ± 42.58 ^#^	99.89 ± 8.35 ^##^
group E	38.10 ± 5.03 ^##^	143.39 ± 17.74 ^##^	48.95 ± 6.24 ^##^	755.50 ± 51.82 ^#^	102.15 ± 11.32 ^##^
group F	41.07 ± 5.37 ^##^	151.88 ± 22.64 ^##^	48.92 ± 6.28 ^##^	776.68 ± 68.20	106.97 ± 15.05 ^#^
group G	41.57 ± 7.43 ^#^	129.50 ± 8.10 ^##^	48.46 ± 6.57 ^##^	769.71 ± 98.94	108.68 ± 9.03 ^#^
group H	36.53 ± 5.32 ^##^	162.25 ± 15.47 ^#^	52.14 ± 7.95 ^##^	792.84 ± 36.84	109.76 ± 10.09
group I	39.70 ± 5.24 ^##^	165.30 ± 19.39 ^#^	53.78 ± 8.57 ^##^	746.88 ± 19.32 ^##^	114.82 ± 7.74

Values are mean ± SD, *n* = 12/group; PI: prostatic index; DHT: dihydrotestosterone; ACP: acid phosphatase; PACP: prostatic acid phosphatase; SRD5A2: type 2,5-alpha-reductase. # *p* < 0.05; ## *p* < 0.01 *vs.* BPH model control group, one way ANOVA and Dunnett’s multiple comparisons *t*.

#### 2.3.4. Effect of *AESS* on Serum PACP Concentration

PACP is a well-known prognostic biochemical indicator for diagnosis and often used to monitor the progression in BPH and prostate cancer. *t* is considered an essential regulator of cell growth and proliferation in the prostate. Generally speaking, PACP serum levels are abnormally elevated in the patients with BPH, when prostate cancer and patients with prostatic inflammatory conditions [28]. As the results in Table 3 show, the mice in the BPH model control group (816.66 ± 60.85 ng/L, *p* < 0.01) exhibited a significant increase compared to the control group (647.85 ± 54.63 ng/L, *p* < 0.01). However, the finasteride-treated group (698.06 ± 32.38 ng/L, *p* < 0.01) and QKPT control group (675.98 ± 55.81 ng/L, *p* < 0.01) decreased the level of PACP in serum more than the BPH group. At the *AESS* group, only sample **E** (769.71 ± 98.94 ng/L), **G** (776.68 ± 68.20 ng/L) and **H** (792.84 ± 36.84 ng/L) showed no significantly lower serum PACP concentration compared with BPH model control group. From the result, the significant increase in serum PACP concentration in the BPH model control group compared to the control group in the mice. Samples **A**–**E** and I significantly reduced the PACP concentration in serum, but the effects were not the same. The reason for this might be because the different producing areas in the sample have different components. 

#### 2.3.5. Effect of *AESS* on Serum SRD5A2 Concentration

Two 5α-reductase isozymes responsible for testosterone converted to DHT in the body and type-1,5-reductase is expressed in the skin and liver and type-2,5-reductase predominates in the prostate, respectively [29]. Then, all mice type-2,5-reductase concentration in serum has been monitored, to evaluate the therapeutic effects of BPH. As the results in Table 3 show, the BPH model control group exhibited significant increases in the levels of SRD5A2 in serum (125.23 ± 9.69 pg/L, *p* < 0.01) compared with the control group (78.23 ± 9.26 pg/L). However, the finasteride control group (90.72 ± 9.54 pg/L, *p* < 0.01) and QKPT control group (89.86 ± 12.60 pg/L, *p* < 0.01) decreased the level of SRD5A2 in serum more than the BPH group. In the *AESS* group, only sample H (400.53 ± 12.81 pg/L) and I (114.82 ± 7.74 pg/L) did not decrease the level of SRD5A2 in serum compared with the BPH group, other samples show significant decreases in the levels of SRD5A2 in serum compared with the BPH group. In the present study, most of the samples showed significantly reduced levels of SRD5A2 in serum except samples **H** and **I**. These findings in combination with the results of the indicators assay suggest that *AESS* is an effective treatment for BPH.

### 2.4. Analysis of Spectrum-Effect Relationship

#### 2.4.1. Results of Grey Relational Analysis

In the present study, the five pharmacodynamics indexes (PI, DHT, ACP, PACP, and SRD5A2) were chosen as five reference series and the 18 values of peak areas were chosen as compared series. Then, the GRD between the compared and reference series was calculated with a resolution ratio of 0.5. The higher the GRD, the greater the effect of anti-benign prostatic hyperplasia. The grey system theory used Grey Modeling software (Grey relational degree V6.0, Nanjing University of Aeronautics and Astronautics, Nanjing, China). The grey relational grade is shown in Table 4. As given in Table 4, the average GRG between the five pharmacodynamics indexes and the 18 values of peak areas and were as follows: A_18_ > A_14_ > A_17_ > A_16_ > A_4_ > A_13_ > A_15_ > A_9_ > A_1_ = A_10_ > A_11_ > A_7_ > A_5_ > A_3_ > A_12_ > A_6_ > A_8_ > A_2_. A_18_ indicated a relatively high influence for anti-benign prostatic hyperplasia, A_14_ and A_17_ showed a noticeable influence for anti-benign prostatic hyperplasia, A_16_, A_4_, A_13_, A_15_, A_9_, A_1_ and A_10_ remained a small influence for anti-benign prostatic hyperplasia, and A_11_, A_7_, A_5_, A_3_, A_12_, A_6_, A_8_ and A_2_ contained a negligible influence for anti-benign prostatic hyperplasia. Then, A_18_, A_17_ and A_14_ were greater than other peaks, which suggested that the three components had marked influence. The peak of the A_18_ is the level with the highest grey relational grade, suggesting that A_18_ common peak of *S. stolonifera* may be the active ingredient for anti-benign prostatic hyperplasia. At the same time, both of A_14_ and A_17_ peaks had a higher grey relational grade than the other compounds, which indicated that the two compositions had a relatively high influence on anti-benign prostatic hyperplasia. Therefore, the three constituents were considered as key components which could play very important roles on bioactivities. However, further statistical analysis process needed to be done to find whether the grey relational grade performed positive correlation or negative correlation on treatment of BHT.

**Table 4:** The calculated grey relational coefficient and grey relational grade for 18 comparability sequences and their order.

NO.	Y1	Y2	Y3	Y4	Y5	Average	Order
A_1_	0.573	0.651	0.584	0.641	0.573	0.604	9
A_2_	0.522	0.525	0.496	0.620	0.522	0.537	17
A_3_	0.557	0.630	0.536	0.640	0.557	0.584	13
A_4_	0.626	0.698	0.595	0.673	0.626	0.644	5
A_5_	0.566	0.621	0.539	0.659	0.566	0.590	12
A_6_	0.532	0.583	0.534	0.641	0.532	0.564	15
A_7_	0.567	0.638	0.564	0.644	0.567	0.596	11
A_8_	0.525	0.587	0.514	0.608	0.525	0.552	16
A_9_	0.619	0.615	0.650	0.636	0.619	0.628	8
A_10_	0.588	0.644	0.546	0.655	0.588	0.604	9
A_11_	0.579	0.581	0.548	0.702	0.579	0.598	10
A_12_	0.555	0.622	0.538	0.646	0.555	0.583	14
A_13_	0.623	0.641	0.611	0.716	0.623	0.643	6
A_14_	0.724	0.733	0.669	0.774	0.724	0.725	2
A_15_	0.631	0.622	0.661	0.621	0.631	0.633	7
A_16_	0.656	0.635	0.650	0.656	0.656	0.651	4
A_17_	0.736	0.658	0.704	0.677	0.736	0.702	3
A_18_	0.869	0.744	0.838	0.819	0.869	0.828	1

#### 2.4.2. Results of Partial Least Squares Regression Analysis

**Figure 3:**
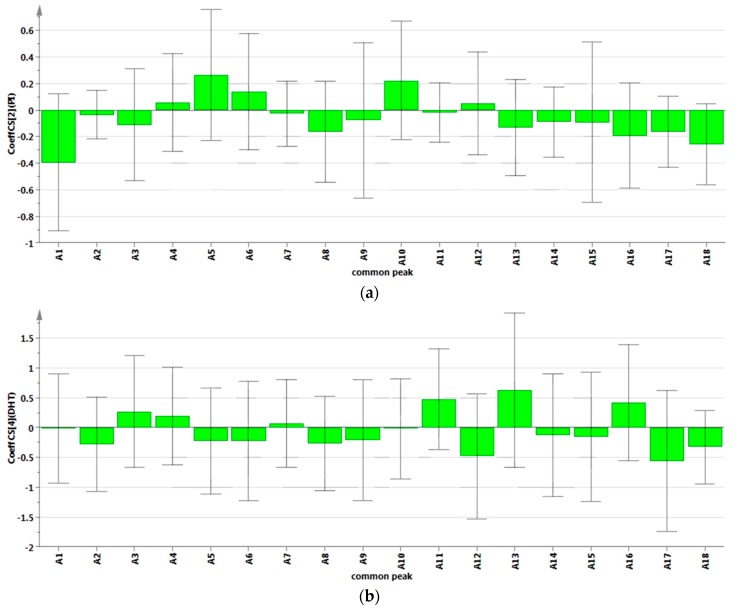

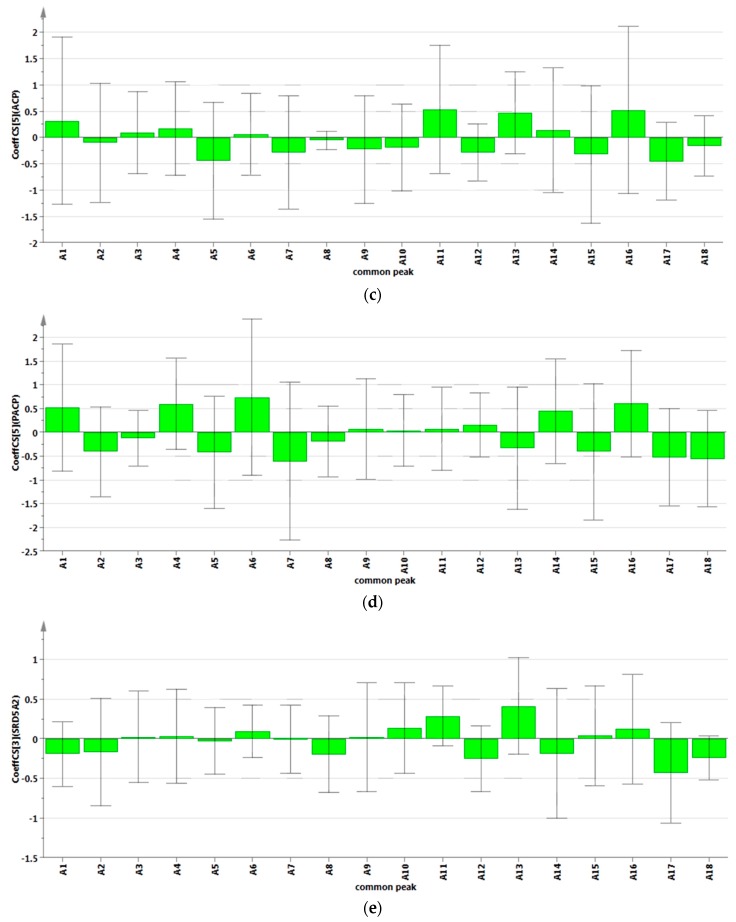
Regression coefficient figure of the 18 compounds and the five indicates about benign prostatic hyperplasia. (**a**) is the regression coefficient figure of PI and the 18 compounds; (**b**) is the regression coefficient figure of DHT and the 18 compounds; (**c**) is the regression coefficient figure of ACP and the 18 compounds; (**d**) is the regression coefficient figure of PACP and the 18 compounds; (**e**) is the regression coefficient figure of SRD5A2 and the 18 compounds.

The relationship between the 18 compounds and the five indicates about BPH were used to build the regression models, the regression coefficient were shown Figure 3. The regression equation obtained for PLS model is given as follows:

Y (PI) = −0.39A_1_ − 0.03A_2_ − 0.11A_3_ + 0.06A_4_ + 0.26A_5_ + 0.14A_6_ − 0.03A_7_ − 0.16A_8_ − 0.08A_9_ + 0.22A_10_ − 0.02A_11_ + 0.05A_12_ − 0.13A_13_ − 0.09A_14_ − 0.09A_15_ − 0.19A_16_ − 0.16A_17_ − 0.25A_18_ (7)

Y (DHT) = −0.01A_1_ − 0.27A_2_ + 0.26A_3_ + 0.19A_4_ − 0.22A_5_ − 0.23A_6_ + 0.07A_7_ − 0.26A_8_ − 0.20A_9_ − 0.02A_10_ + 0.48A_11_ − 0.48A_12_ + 0.63A_13_ − 0.12A_14_ − 0.15A_15_ + 0.42A_16_ − 0.56A_17_ − 0.33A_18_ (8)

Y (ACP) = 0.32A_1_ − 0.10A_2_ + 0.09A_3_ + 0.16A_4_ − 0.44A_5_ + 0.06A_6_ − 0.28A_7_ − 0.06A_8_ − 0.22A_9_ − 0.19A_10_ + 0.53A_11_ − 0.28A_12_ + 0.47A_13_ + 0.13A_14_ − 0.32A_15_ + 0.52A_16_ − 0.45A_17_ − 0.16A_18_ (9)

Y (PACP) = 0.52A_1_ − 0.41A_2_ − 0.12A_3_ + 0.59A_4_ − 0.42A_5_ + 0.74A_6_ − 0.61A_7_ − 0.19A_8_ + 0.07A_9_ + 0.04A_10_ + 0.07A_11_ + 0.16A_12_ − 0.33A_13_ + 0.45A_14_ − 0.41A_15_ + 0.61A_16_ − 0.52A_17_ − 0.55A_18_ (10)

Y (SRD5A2) = −0.19A_1_ − 0.17A_2_ + 0.02A_3_ + 0.04A_4_ − 0.03A_5_ + 0.09A_6_ − 0.01A_7_ − 0.19A_8_ + 0.02A_9_ + 0.13A_10_ + 0.29A_11_ − 0.25A_12_ + 0.41A_13_ − 0.18A_14_ + 0.04A_15_ + 0.12A_16_ − 0.43A_17_ − 0.24A_18_ (11)

Equations (7)–(11) were the regression models of 18 common peaks area values and mice serum ACP activity, PI, and the concentration of serum DHT, PACP and SRD5A2, respectively. Equation (7) and Figure 3a shows that A_1_–A_3_, A_7_, A_8_, A_11_, A_13_–A_18_ were in positive correlation with IP. However, A_4_–A_6_, A_9_, A_10_, A_12_ and A_13_ showed negative correlation with reduced IP. We extracted two principal components *R*^2^ = 0.9090, which indicated that the regression model had 0.9090 explanatory power for reduced PI, which indicated that the model has high precision. A_1_, A_18_, A_8_ and A_17_ were higher correlation compared with other compounds, which denotes that the four compounds have good effect in reduced PI. Y (DHT) and Figure 3b is the regression model of serum DHT concentration and its regression coefficient figure, respectively. From Equation (8) and Figure 3b, A_1_, A_2_, A_5_, A_6_, A_8_–A_10_, A_12_, A_15_, A_17_ and A_18_ were in positive correlation with a reduction in the concentration of serum DHT, when three principal components *R*^2^ = 0.8321 were extracted. This indicated that the regression model prediction accuracy was satisfactory. As shown in Equation (9) and Figure 3c, the model has high explanatory power for reduced mice serum ACP activity (*R*^2^ = 0.7659), when three main components have been extracted. A_17_, A_5_, A_15_ and A_7_ showed a greater reduction in serum ACP activity than other common peaks, which denotes that this compound may be one of the main compounds in reduced serum ACP activity. Equation (9) and Figure 3c were the regression equation and regression coefficient figure obtained for the PLS model, when three principal components *R*^2^ = 0.8271 were extracted. As Equation (10) and Figure 3d show, A_7_ is the most important property to describe the anti-prostate hyperplasia activity followed byA_18_, A_17_, A_5_, A_2_, A_15_, A_13_, A_8_ and A_3_, and all these common peaks were directly correlated with a reduction in the concentration of serum PACP. The remained compounds were shown to have inverse correlation. Equation (11) and Figure 3e show the PLS regression equation and regression coefficient figure when three principal components were extracted. For *R*^2^ = 0.8273, the model has high explanatory power for reduced mice serum SRD5A2 concentration. Y (SRD5A2) is directly correlated with A_17_, A_12_, A_18_, A_1_, A_2_, A_14_, A_8_, A_5_ and A_7_. An inverse correlation is observed between A_13_, A_11_, A_10_, A_16_, A_6_, A_4_, A_3_ and A_9_. The results showed that increasing A_17_, A_12_, A_18_, A_1_, A_2_, A_14_, A_8_, A_5_ and A_7_ and reducing A _13_, A_11_, A_10_, A_16_, A_6_, A_4_, A_3_ and A_9_ peak area can reduce the mice serum SRD5A2 concentration.

## 3. Experimental Section

### 3.1. Materials

Twenty-four batches of *S. stolonifera* samples from various sources (Table 1) were authenticated by Deyuan Chen (Guiyang College of TCM, Guiyang, China). One hundred grams of *S. stolonifera* was macerated in 1000 mL of water for 30 min and decocted with water three times (3 h each time). The filtrates from each decoction were blended and concentrated to a thick solution using a rotary evaporator, the conditions of concentrating the extract were 60 °C, 20 rpm, and −0.09 MPa. The concentrated sample was dried in vacuum oven and so did the dried powders. All of the 24 batches of *S. stolonifera* were decocted and dried with the same procedure. The dried powders was weighed and stored in a sealed container in a refrigerator at a temperature of −20 ± 2 °C until use. Methanol (MeOH) of chromatographic grade was purchased from Tedia Chemicals (Faireld, OH, USA), as well as the HPLC grade phosphoric acid with a purity of 99% (Houston, TX, USA). Finasteride was obtained from Merck (Hangzhou, China). Testosterone propionate was manufactured by Shanghai GM Pharmaceutical Co., Ltd. (Shanghai, China). Qianlie Kang Pule’an Tablet was obtained from Zhejiang Conba Pharmaceutical (Lanxi, China), each individual Qianlie Kang Pule’an Tablet (QKPT) consists of 0.5 g of the *Brassica campestris* L. pollen without any additional ingredients. In the present study all the enzyme-linked immunosorbent assay (ELISA) kits were obtained from Shanghai MLBIO Biotechnology Co., Ltd (Shanghai, China). All other chemicals and solvents used were of analytical grade.

One hundred and fifty-six adult Chinese KM male mice, Specific pathogen-free (SPF, Certificate No. SCXK 2014-0011) grade, weighing 18–22 g were purchased from Changsha Tianqi Biotechnology Co., Ltd. (Changsha, China) for this study. The mice were acclimatized to laboratory environment (20–25 °C) with a 12 h light-darkness cycle for 3 days prior to experimentation. Temperature, humidity, and light conditions in the mice environment were kept constant, with food and water provided ad libitum. Animal care and experiments were conducted in accordance with the guidelines of the Chinese Council on Animal Care and approved by the Guizhou Normal University Animal Care and Use Committee.

### 3.2. Instruments

HPLC fingerprints of *AESS* from 24 different regions samples were performed using Thermo Scientific DIONEX UltiMate 3000 system (Waltham, MA, USA), consisting of binary solvent delivery pump, auto sampler manager, column compartment, photo diodearray detector and Chromeleon 7.1 ChemStation (Thermo Scientific, Waltham, UK). The weighing was done with AL204 1/10,000 electronic analytical balance and XS-105DU 1/100,000 electronic analytic balance from Mettler Toledo Instruments (Shanghai) Co., Ltd. (Shanghai, China). The levels of type II 5-alpha-reductase (SRD5A2), acid phosphatase (ACP), prostatic acid phosphatase (PACP) and DHT in serum were measured by Spectra Max 484 Molecular Devices Co., Ltd. (New York, NY, USA).

### 3.3. Determination of HPLC Fingerprints

#### 3.3.1. Chromatographic Separation

Chromatographic separations were carried out on a Diamonsil C18 column (4.6 mm × 250 mm, 5 μm), column temperature was set at 30 °C. The mobile phase consisted of methanol (A) and water (containing 0.05% phosphoric acid) with the following gradient elution: 0–5 min, 99% B; 5–8 min, 99%–98% B; 8–19 min, 98%–87% B; 19–22 min, 87%–85% B; 22–35 min, 85%–80% B; 35–38 min, 80%–70% B; 38–53 min, 70%–65% B; 53–63 min, 65%–60% B; 63–73 min, 60%–55% B; 73–83 min, 55%–50% B; 83–93 min, 50%–15% B. Flow rate was 0.7 mL/ min^−1^ and an injection volume of 20 μL. The DAD detection wavelength was set at 256 nm and temperature of the auto-sampler was maintained at 30 °C.

#### 3.3.2. Solution’s Preparation

An equivalent to 2.0 g of dry *S. stolonifera* extracts powder was accurately weighed and fully dissolved into 24 mL water. The extracted solution was filtered through a 0.45 μm micropore film. All of the 24 batches of *S. stolonifera* extracts powder were prepared with the same procedure for HPLC fingerprint analysis.

#### 3.3.3. Similarity Analysis (SA)

Twenty-four batches of *S. stolonife*ra collected from various locations were analyzed under optimal conditions, and matched automatically by professional software named Similarity Evaluation System (SES) for Chromatographic Fingerprint of Traditional Chinese Medicine, composed by Chinese Pharmacopoeia Committee (Version 2004 A; Beijing, China). Then, the reference chromatograms were generated by this system using the Median method from the general comparison of the chromatograms of 24 batches of *S. stolonifera* extracts. The similarities between the entire chromatographic profiles of 24 batches of *S. stolonifera* and the reference chromatogram were evaluated by SES software. The differences of correlation coefficients indicated variation of the fingerprint and internal qualities of these samples.

#### 3.3.4. Hierarchical Clustering Analysis (HCA)

HCA is a way of grouping pattern vectors which is used to find relatively homogeneous clusters of cases based on measured characteristics. This technique starts with each case in a separate cluster and then combines the clusters sequentially, reducing the number of clusters at every step until all the objects or sample clustered into one category. The similarity or dissimilarity between samples (objects) is usually represented as a tree or dendrogram for ease of interpretation [30]. In this study, the HCA of samples was performed by SPSS software (SPSS for Windows20.0, SPSS Inc., Armonk, NY, USA).

#### 3.3.5. Principal Component Analysis

In many cases, a number of variables need to be analyzed to achieve a comprehensive evaluation. Therefore, date decompositions should be conducted to reduce multidimensional data sets to lower dimensions. Among these techniques, Principal component analysis (PAC) is a very useful tool of data processing for data compression and information extraction which visualizes the main relationships that exist among a large number of variables in terms of a smaller number or potential factors without losing much information by extracting data, removing redundant information, and highlighting hidden features [31]. Here, SPSS computer software (SPSS for Windows20.0, SPSS Inc.) was used to evaluate the differences among the 24 samples by analyzing the relative 18 common peaks.

#### 3.3.6. Screening for Differences between Samples

Based on the chromatographic fingerprints, those samples with significant variations in chemical profiles were selected to investigate their anti-benign prostatic hyperplasia bioactivities as well as profile-effect correlations.

### 3.4. Anti-Benign Prostatic Hyperplasia Experimentation

#### 3.4.1. Castration Procedure

To exclude the influence of intrinsic testosterone, all mice but twelve were anesthetized by inhalation of isoflurane and castrated after intramuscular administration of penicillin (7.14 × 104 IU/kg body weight). Castration was performed by removing the testicles and epididymal fat through the scrotal sac, according to the method published previously [32].

#### 3.4.2. Induction of BPH and Treatments

In the present study, the mice were randomly divided into 13 groups (*n* = 12 each) as follows: (1) the control group, which received NS administered orally and placebo injections of the olive oil injected subcutaneously (s.c.); (2) BPH model control group, which received NS administered orally and testosterone propionate (TP) (7.5 mg/kg body weight, s.c.); (3) positive control group, which received finasteride (1 mg/kg body weight) administered orally and TP (7.5 mg/kg body weight, s.c.); (4) Qianlie Kang Pule’an Tablet (QKPT) control group, which received QKPT (750 mg/kg body weight) administered orally and TP (7.5 mg/kg body weight, s.c.); (5–13) nine samples (from the results of screening differences samples) of *AESS* (equivalent to 4 g dry *S. stolonifera*/kg body weight) orally administered and TP (7.5 mg/kg body weight, s.c.). All mice were treated once a day for two weeks. Body weight was measured once the three days during the experiment. The application volume was calculated in advance, based on the most recent recorded body weight of individual animals. At the end of the experimental period, mice were fasted for 12 h after administration of last dose. Blood samples were drawn from the retro-orbital blood vessels and then the mice were euthanized. The prostate gland was freed from connective tissues, excised and weighed. The prostate organs were immediately fixed in 10% buffered formaldehyde solution and stored at −20 °C for histological analysis.

#### 3.4.3. Determination of Prostatic Index (PI)

Prostate weight (PW) to body weight (BW) ratio of the mice in each group was calculated. The PI was calculated as: PW/BW × 100. The mean PI ratios were calculated of each group.

#### 3.4.4. Immunohistochemical Analysis

All the blood samples were centrifuged at 5 kg for 10 min at 4 °C to obtain serum for determination of DHT, ACP, PACP and SRD5A2 by enzyme-linked immunosorbent assay (ELISA) kits. Test was carried out according to the manufacturer’s instructions. Values were expressed as per L in serum (Table 3).

#### 3.4.5. Statistical Analysis

Data were expressed as means ± standard deviation (SD) values. Statistical analysis of the data was assessed using analysis of variance (ANOVA) followed by the Dunnett’s multiple comparison test, using SPSS computer software Version 20 (New York, NY, USA). The levels of significance were set at *p* < 0.05, *p* < 0.01.

### 3.5. Spectrum-Effect Relationship Analysis

#### 3.5.1. Grey Relational Analysis

Grey relational analysis (GRA) is an important branch of grey system theory which has been successfully applied to solve many concrete real-world problems that have complicated interrelationships between multiple factors and variables [33]. Overall, the basic principle of the GRA is analyze the degree of approximation of the factors and variables in large the data sets when there is insufficient information, and according to the analysis result estimate correlation of factors and variables [34]. Then, GRA can help to compensate for the shortcomings in statistical regression when experiments are ambiguous or when the experimental method cannot be carried out exactly. In view of this, GRA was used to analysis the spectrum-activity relationships between chemical fingerprint and anti-benign prostatic hyperplasia bioactivity of the *AESS*, in present study.

#### 3.5.2. Partial Least Squares Regression

Partial least squares regression (PLSR) is a frequently applied technique that specifies a linear relationship between a set of dependent variables from a large set of independent variables, especially when the sample size is small relative to the dimension of these variables. It was originally proposed in economics and chemo metrics as an alternative approach to OLS in ill-conditioned linear regression problems. However, now, it has been successfully extended to other scientific areas, such as bioinformatics, economics, and medicine, *etc.* [35,36]. In this study, PLSR was used to model the correlation between 18 common peaks (predictor variables) and the five indicates (response variable) of anti-benign prostatic hyperplasia, respectively. The PLSR modeling was performed using software Simca-p (Simca-p13.0, Umetrics, Umeå, Sweden). More detailed description of the PLSR technique can be found in [37,38].

## 4. Conclusions

In this study, the chemical fingerprints of 24 batches of *S. stolonifera* sample from various sources were established by HPLC-DAD. Then, the anti-benign prostatic hyperplasia effects of these samples were determined, using a testosterone-induced BPH mouse model. With the help of GRA and PLSR, the relationship between the fingerprints and efficacy of *S. stolonifera* was elucidated. The results showed that the 24 batches of *S. stolonifera* from different habitats, harvest season and processing methods may lead to different quality and pharmacological activity. Under this experimental condition, greater relatively differences in mice with PI, DHT, PACP SRD5A2 and ACP of these nine batches of *S. stolonifera* has been proved from different resources. From the result of GRA, considering the five indexes (PI, DHT, ACP, PACP and SRD5A2) of BPH, A_14_, A_17_ and A_18_ showed relatively higher GRA than other common peaks. PLSR results were shown in Equations (7)–(11) and Figure 3. A_2_, A_8_, A_17_ and A_18_ all showed negative correlation with these five indicators, but A_4_ was positively correlated with all the five indicators. Besides, A_1_, A_3_, A_5_, A_6_, A_7_, A_9_, A_10_, A_11_, A_12_, A_13_, A_14_, A_15_ and A_16_ can reduce some of these indicators. According to the result of GRA and PLSR, the strength of anti-benign prostatic hyperplasia bioactivity *AESS* was mainly affected by A_14_ (Chlorogenic acid), A_17_ (Quercetin 5-*O*-β-d-glucopyranoside) and A_18_ (Quercetin 3-*O*-β-l-rhamnopyranoside). That is to say, chlorogenic acid, quercetin 5-*O*-β-d-glucopyranoside and quercetin 3-*O*-β-l-rhamnopyranoside might be the main active components affecting anti-benign prostatic hyperplasia bioactivity of *S. stolonifera*. Based on the studies above, further study is needed to investigate these three components in the anti-prostate hyperplasia activities. The investigation of HPLC fingerprints and anti-prostate hyperplasia activities relationships of *S. stolonifera* based on HPLC, GRA, and PLSR could provide a tool to evaluate the differences of internal quality and anti-prostate hyperplasia activities of *S. stolonifera*, providing a sound experimental foundation and model for its study.
